# Sialylation of Human Natural Killer (NK) Cells Is Regulated by IL-2

**DOI:** 10.3390/jcm9061816

**Published:** 2020-06-11

**Authors:** Philip Rosenstock, Kaya Bork, Chiara Massa, Philipp Selke, Barbara Seliger, Rüdiger Horstkorte

**Affiliations:** 1 Institute for Physiological Chemistry, Martin-Luther-University Halle-Wittenberg, Hollystr. 1, D-06114 Halle (Saale), Germany; kaya.bork@medizin.uni-halle.de (K.B.); Philipp.Selke@uk-halle.de (P.S.); ruediger.horstkorte@medizin.uni-halle.de (R.H.); 2 Institute for Medical Immunology, Martin-Luther-University Halle-Wittenberg, Magdeburger Str. 2, D-06112 Halle (Saale), Germany; chiara.massa@medizin.uni-halle.de (C.M.); barbara.seliger@uk-halle.de (B.S.)

**Keywords:** NK cells, sialylation, sialyltransferases, interleukin-2, GD3, lectins, polySia, NCAM, CD56, NK-92

## Abstract

Sialic acids are terminal sugars on the cell surface that are found on all cell types including immune cells like natural killer (NK) cells. The attachment of sialic acids to different glycan structures is catalyzed by sialyltransferases in the Golgi. However, the expression pattern of sialyltransferases in NK cells and their expression after activation has not yet been analyzed. Therefore, the present study determines which sialyltransferases are expressed in human NK cells and if activation with IL-2 changes the sialylation of NK cells. The expression of sialyltransferases was analyzed in the three human NK cell lines NK-92, NKL, KHYG-1 and primary NK cells. NK-92 cells were cultured in the absence or presence of IL-2, and changes in the sialyltransferase expression were measured by qPCR. Furthermore, specific sialylation was investigated by flow cytometry. In addition, polySia and NCAM were measured by Western blot analyses. IL-2 leads to a reduced expression of *ST8SIA1*, *ST6GAL1* and *ST3GAL1*. α-2,3-Sialylation remained unchanged, while α-2,6-sialylation was increased after IL-2 stimulation. Moreover, an increase in the amount of NCAM and polySia was observed in IL-2-activated NK cells, whereas GD3 ganglioside was decreased. In this study, all sialyltransferases that were expressed in NK cells could be identified. IL-2 regulates the expression of some sialyltransferases and leads to changes in the sialylation of NK cells.

## 1. Introduction

**Figure 1:**
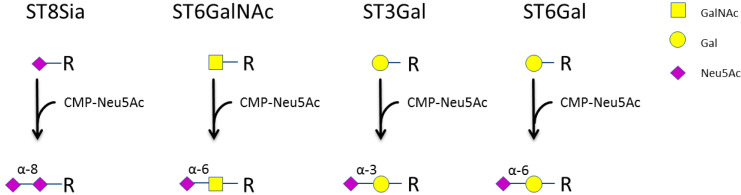
Groups of human sialyltransferases. Sialyltransferases catalyze the transfer of N-acetylneuraminic acid (Neu5Ac) to N-acetylgalactosamine (GalNAc), galactose (Gal) or other Neu5Ac molecules.

All cells of the human body, including immune cells, are glycosylated and glycan structures are crucial for different immune functions [1,2]. Sialic acids are located at the outside of cells as terminal sugars of N- and O-glycans and glycosphingolipids. They are comprised of a 9-carbon backbone and are derivatives of neuraminic acid. The almost only sialic acid that is synthesized in humans is N-acetylneuraminic acid (Neu5Ac) [3,4]. Its synthesis occurs in the cytosol, and it further reacts with cytidine triphosphate in the nucleus to form cytidine monophosphate (CMP)-Neu5Ac [5]. CMP-Neu5Ac is then transported to the Golgi, where different sialyltransferases catalyze the attachment to other sugar molecules [6]. In humans, 20 different sialyltransferases are expressed, which can be classified into four groups (Figure 1). α-2,8-sialyltransferases (ST8Sia; EC 2.4.99.8) catalyze the attachment of Neu5Ac to other sialic acids via α-2,8-linkage. GalNAc α2,6-sialyltransferases (ST6GalNAc; EC 2.4.99.3) are responsible for the attachment of Neu5Ac to N-acetylgalactosamine (GalNAc). Moreover, the β-galactoside α-2,6- and β-galactoside α-2,3-sialyltransferases (ST6Gal, EC 2.4.99.1; ST3Gal, EC 2.4.99.4) catalyze the attachment of Neu5Ac to galactose (Gal) via α-2,6 or α-2,3-linkage [7]. Even though the sialyltransferases from one group catalyze the same reaction, they have different substrate specificities [8,9]. A detailed list of the 20 sialyltransferases can be found in Supplementary Material Table S1.

As sialic acids are located at the end of glycan structures, they are involved in cell adhesion and cell-cell interaction [10]. Since all human cells have sialic acids on their surface, sialylation plays also an important role for the immune system to distinguish between healthy and altered cells [11]. Important effector cells of the innate immune system are natural killer (NK) cells. NK cells are large granular lymphocytes that represent 10–15% of the blood lymphocytes and are able to kill virus-infected cells and tumor cells [12]. Discrimination between infected or mutated cells and normal cells is mediated via different activating or inhibitory receptors on NK cells that bind ligands on other cells [13]. In general, two main NK cell subsets can be defined based on the surface expression of the neural cell adhesion molecule (NCAM) or CD56. Cells with a lower NCAM/CD56 expression (CD56^dim^) have a high cytotoxic activity, while the main function of NK cells with a higher NCAM/CD56 expression (CD56^bright^) is the production of immunoregulatory cytokines [14]. NK cells require different cytokines to become fully activated. One of these cytokines is interleukin-2 (IL-2), which is produced by T cells in vivo [15]. Upon IL-2 stimulation NK cells express and secrete more interferon-γ (IFN-γ) and have a higher cytotoxic potential [16,17]. Moreover, the expression of different NK cell receptors is altered after IL-2 activation leading to a change in the receptor repertoire and the composition of the NK cell surface [18,19,20].

As all human cells, NK cells are sialylated, and lectin staining could reveal that CD56^bright^ NK cells have a higher degree of sialylation than CD56^dim^ NK cells [21]. A special form of sialylation is found on NCAM/CD56. This molecule has long chains of sialic acid molecules attached to each other via α-2,8-linkage, which is called polysialic acid (polySia). ST8Sia IV is responsible for the polySia synthesis in NK cells [22]. Moreover, NK cells express sialic-acid-binding immunoglobulin-like lectins (Siglecs), special receptors that are capable of binding sialic acids. Siglec-7 and Siglec-9, which are inhibitory receptors, are expressed on NK cells and help to identify healthy autologous cells. However, many tumor cells express ligands for these Siglecs and can therefore escape the killing by NK cells [23]. Siglecs can not only bind to sialic acids on other cells but also to sialic acids on the same cell. This interaction of Siglecs with sialic acids on the same cell is termed masking and might regulate the activity of the receptor as the binding site is blocked [24,25].

Most of the studies regarding sialic acids and NK cells are focused on Siglecs on NK cells and their binding of sialylated tumor cells. The sialylation of NK cells, which could play a role in cell adhesion, cell–cell interaction and masking of Siglecs, is so far poorly understood. To gain more knowledge about sialic acids on NK cells, we analyzed which sialyltransferases are constitutively expressed in both human NK cell lines and primary human NK cells. Our experiments further show that the expression of sialyltransferases as well as the sialylation of NK cells is influenced by their activation with IL-2.

## 2. Experimental Section

### 2.1. Cells and Cell Culture

The human NK cell lines NK-92, NKL and KHYG-1 were kindly provided by Roland Jacobs (Hannover Medical School, Hannover, Germany). The cells were cultured in RPMI 1640 (Lonza, Basel, Switzerland) supplemented with 10% fetal calf serum (FCS) (GE Healthcare, Little Chalfont, UK), 100 U/mL penicillin and 100 mg/mL streptomycin (Thermo Fisher Scientific, Waltham, MA, USA) and 1 mM sodium pyruvate (Thermo Fisher Scientific) in humidified 5% CO_2_ atmosphere at 37 °C. For standard cultivation, the medium was supplemented with 200 U/mL of recombinant human IL-2 (Novartis Pharma GmbH, Zwickau, Germany). For the experiments, cells were washed 4 times with PBS and incubated in medium without IL-2 for 24 h. Cells were either directly used (control) or stimulated with 1000 U/mL IL-2 for 4 h or 24 h, respectively. Primary human NK cells were isolated from the buffy coats of healthy blood donors (1 male / 2 females) using density gradient centrifugation followed by negative selection using the Mojo Human NK cells kit (Biolegend, San Diego, CA, USA). The purity of NK cells was checked with flow cytometry by CD3, CD56 and CD16 staining and was over 90% for all samples.

### 2.2. PCR and Quantitative Real-Time PCR Analysis

RNA was isolated from human NK cell lines and primary NK cells using the Quick-RNA™ MiniPrep Kit (Zymo Research, Irvine, CA, USA) according to the manufacturer’s instructions. Quality and concentration of the RNA was analyzed using the NanoDrop 1000 Spectrophotometer (Thermo Fisher Scientific). RNA (2 μg) was transcribed into cDNA using SuperScript™ II Reverse Transcriptase according to the manufacturer’s instructions. PCR reactions were performed using DreamTaq DNA polymerase (Thermo Fisher Scientific), and products were separated on a 1.5% agarose gel. The following conditions were used: initial denaturation for 2 min at 95 °C, 35 cycles (30 s at 95 °C, 30 s at 55 °C, 30 s at 72 °C), final elongation for 5 min at 72 °C. Primer pairs for all sialyltransferases are listed in Table 1. The primer pairs are always separated by at least one intron on the corresponding genomic DNA and were carefully checked for possible mismatches in silico. Additionally, randomly selected PCR fragments (e.g., *ST8SIA1* and *ST8SIA4* for NK-92 cells) were sequenced as a control.

The sialyltransferase expression after IL-2 stimulation was measured via quantitative real-time PCR (qPCR) using the iQ™ 5 Multicolor Real-Time PCR Detection System (Biorad, Hercules, CA, USA) and qPCR GreenMaster (Jena Bioscience, Jena, Germany) with the same primer pairs used for normal PCR. NK-92 cells were incubated without IL-2 for 24 h. Cells were either left untreated or treated with 1000 U/mL IL-2 for 4 h prior to RNA isolation and cDNA synthesis. The following conditions were used for qPCR: initial denaturation for 1:30 min at 95 °C, 40 cycles (10 s at 95 °C, 10 s at 62 °C, 25 s at 72 °C), final elongation for 1 min at 72 °C followed by a melting curve analysis. Quantification was performed according to Pfaffl et al. [26] as:

(1) $$ratio=\frac{{\left(E_{target}\right)}^{\Delta Ct_{target}\left(control-sample\right)}}{{\left(E_{reference}\right)}^{\Delta Ct_{reference}\left(control-sample\right)}}$$

The amplification efficiency (E) was directly calculated from the amplification curves using the LinReg Software Version 2018.0 [27]. Beta-2 microglobulin (*B2M*) was used as a reference gene to normalize the data.

### 2.3. Flow Cytometry Analysis

NK-92 cells were incubated without IL-2 for 24 h and either directly analyzed or treated with 1000 U/mL IL-2 for additional 24 h. Cells were washed with PBS and resuspended in ice-cold staining buffer (PBS supplemented with 5% bovine serum albumin and 0.05% NaN_3_). A total of 300,000 cells per tube were stained with monoclonal anti-GD3 antibody (ab11779, Abcam, Cambridge, UK) at 1:50 dilution or with biotinylated Sambucus Nigra Lectin (SNA, 20 μg/mL) or biotinylated Maackia Amurensis Lectin II (MAL II, 10 μg/mL) (both from Vector Laboratories, Burlingame, CA, USA) for 30 min at 4 °C. The cells were washed twice with staining buffer and incubated with FITC-labeled goat anti-mouse secondary antibody (Thermo Fisher Scientific) for GD3 staining or with DyLight 488-labeled streptavidin (Vector Laboratories) for the lectin staining for 30 min at 4 °C.

After two washing steps with staining buffer, the cells were analyzed with a BD Accuri C6 flow cytometer (BD Biosciences, Franklin Lakes, NJ, USA). A total of 10,000 events were collected, and the mean fluorescence intensity (MFI) was measured in the FL1 channel (excitation 488 nm, 533/30 band pass filter). Unstained cells and cells stained only with secondary antibody or streptavidin served as a control. The gating strategy is shown in the Supplementary Material Figure S1.

**Table 1:** Primer pairs.

Gene	Product	Primer
*ST8SIA1*	334 bp	forward: AATCCCAGCATAATTCGGCAAAG
		reverse: AGAAGGGCCAGAAGCCATAG
*ST8SIA2*	340 bp	forward: TCTTCGATCGAGACAGCACCA
		reverse: CACAGGATGCTGCCATTGAGG
*ST8SIA3*	323 bp	forward: ATTTGGCGCTTTCCGTTTGG
		reverse: GCAACATGTCAACAGGTACTGG
*ST8SIA4*	303 bp	forward: TCTAGCTCCTGTGGTGGAGTT
		reverse: TTGGTCAGCCAGTAACCTCTG
*ST8SIA5*	328 bp	forward: AGTCTACTCTGTCCAGGTGCT
		reverse: ACAGTGACCACATCCGTCTTC
*ST8SIA6*	342 bp	forward: GTAACCTACCCCCAACCACAG
		reverse: TCATCAAGCCGGTGGACAAG
*ST6GALNAC1*	356 bp	forward: AGCCTCGGTGGGATTTTGAG
		reverse: GGAGTTGTTCAGGATGCCCC
*ST6GALNAC2*	303 bp	forward: TTTGCCCTGTACTTCTCGGC
		reverse: GGAGGCGATGACTTGGTGAG
*ST6GALNAC3*	339 bp	forward: TGGCCTGCATCCTGAAGAGAA
		reverse: CTTTGGTGGGGGCATTGTTC
*ST6GALNAC4*	299 bp	forward: CGTGGTCTATGGGATGGTCAG
		reverse: TGGAGTGTGATGGCTTGGGA
*ST6GALNAC5*	302 bp	forward: AGGGCACCGTGTTCATCTTC
		reverse: GTGATTGGGATCCCTGCAGAA
*ST6GALNAC6*	339 bp	forward: CGGTCAGCAGTGTTCGTGA
		reverse: GCGGTAGGTGGTCTTGTTGC
*ST6GAL1*	332 bp	forward: TCCCAAAGTGGTACCAGAATCC
		reverse: CTTCTCATAGAGCAGCGGGT
*ST6GAL2*	336 bp	forward: TCTGCTCCTACACGTGGTTATG
		reverse: AGAAGATGGTGGGTTTGGTTGA
*ST3GAL1*	321 bp	forward: ATGTTGGGACCAAGACCACC
		reverse: ACAAGTCCACCTCATCGCAG
*ST3GAL2*	333 bp	forward: CAGATAGTGCCTGGCGAGAA
		reverse: CACTGGGGCGTAGGTGAATC
*ST3GAL3*	337 bp	forward: CCTTTCGCAAGTGGGCTAGA
		reverse: AGAGAATCGCGCTCGTACTG
*ST3GAL4*	335 bp	forward: CCTACAACAAGAAGCAGACCATTC
		reverse: CTGGATCTCGGCTCCATAAGAG
*ST3GAL5*	333 bp	forward: AACAGTGCACCAGTTGAGGG
		reverse: GCCCCAGAACCTTGACTGAG
*ST3GAL6*	297 bp	forward: GACCTCAAGAGTCCTTTGCAC
		reverse: TTCACAGAAATTAAGCTGGTGGTT
*IFN-γ*	330 bp	forward: GAATTGGAAAGAGGAGAGTGACA
		reverse: TACTGGGATGCTCTTCGACCT
*B2M*^1^	86 bp	forward: TGCTGTCTCCATGTTTGATGTATCT
		reverse: TCTCTGCTCCCCACCTCTAAGT

^1^ Primer from Vandesompele et al. [28]

### 2.4. Western Blot Analysis

NK-92 cells were incubated without IL-2 for 24 h and analyzed directly or treated with 1000 U/mL IL-2 for additional 24 h. Cells were washed with PBS and pellets were resuspended in solubilization buffer containing 150 mM NaCl, 50 mM Tris, 1% Triton X, 100 mM phenyl-methylsulfonyl fluoride and a protease inhibitor cocktail (Sigma-Aldrich, St. Louis, MO, USA) at pH 8. After 2 h incubation at 4 °C the samples were centrifuged at 16,000× *g* for 10 min and the supernatant was collected. Proteins were separated by SDS-PAGE on a 10% acrylamide gel and transferred to a nitrocellulose membrane. Protein bands were visualized using ponceau red staining solution containing 0.1% Ponceau S (Carl Roth, Karlsruhe, Germany), 3% trichloroacetic acid and 3% sulfosalicylic acid. The membrane was blocked with 5% milk, and polySia was detected using the monoclonal 735 anti-polySia antibody (a kind gift of Prof. Gerardy-Schahn, Hannover Medical School, Germany) at a concentration of 0.1 μg/mL. For the detection of NCAM/CD56, samples were incubated with endo-N-acetylneuraminidase E (endoNE) (a kind gift of Prof. Gerardy-Schahn) at a concentration of 1 μg/mL at 37 °C for 30 min before gel loading. NCAM/CD56 was detected using monoclonal anti NCAM/CD56 antibody (123C3, Abcam) at a 1:1000 dilution. After incubation with a peroxidase-conjugated secondary antibody, binding could be visualized using the Luminata Forte Western HRP-Substrate (Merck Millipore, Billerica, USA). Images were taken with ChemiDoc MP Imaging System (Biorad) and analyzed with the associated Image Lab software. Ponceau staining served as loading control and was used to normalize the band intensity.

### 2.5. Statistical Analysis.

Data are presented as bar diagrams including mean ± standard deviation (SD). Statistical analyses were performed using OriginPro 2017 software (OriginLab Corporation, Northampton, MA, USA). A difference between untreated and IL-2 treated samples at *p* < 0.05 was considered as statistically significant, and significant *p*-values are displayed on each graph.

## 3. Results

### 3.1. Expression of Sialyltransferases in Human NK Cells

To study sialylation of NK cells, it is important to know which sialyltransferases are expressed. Therefore, RNA was isolated from the human NK cell lines NK-92, NKL and KHYG-1, and cDNA was synthesized. PCR reactions were established to analyze the expression of all 20 different sialyltransferases. The products were then separated on 1.5% agarose gels. Furthermore, RNA was isolated from primary human NK cells derived from 3 healthy blood donors and was also analyzed. The results are summarized in Table 2 (see Supplementary Figure S2 for examples).

There exists a heterogeneous expression of sialyltransferases in NK cell lines and primary NK cells. A total of 11 out of the 20 human sialyltransferases were expressed in the NK cell lines. These include *ST8SIA1*, *ST8SIA4*, *ST8SIA6*, *ST6GALNAC4*, *ST6GALNAC6*, *ST6GAL1* and *ST3GAL1-5*. Interestingly, NKL cells do not express NCAM/CD56 [29] but do express the sialyltransferase *ST8SIA4* that usually synthesizes polySia on NCAM/CD56. No polySia and NCAM/CD56 could be detected in the NKL cell line by Western blot analysis. All primary NK cells express the same sialyltransferases as the NK cell lines. Additionally, NK cells from 2 of the 3 donors also showed an expression for *ST6GAL2* and *ST3GAL6* (Table 2).

### 3.2. ST8SIA1, ST6GAL1 and ST3GAL1 Are Downregulated after IL-2 Stimulation

**Figure 2:**
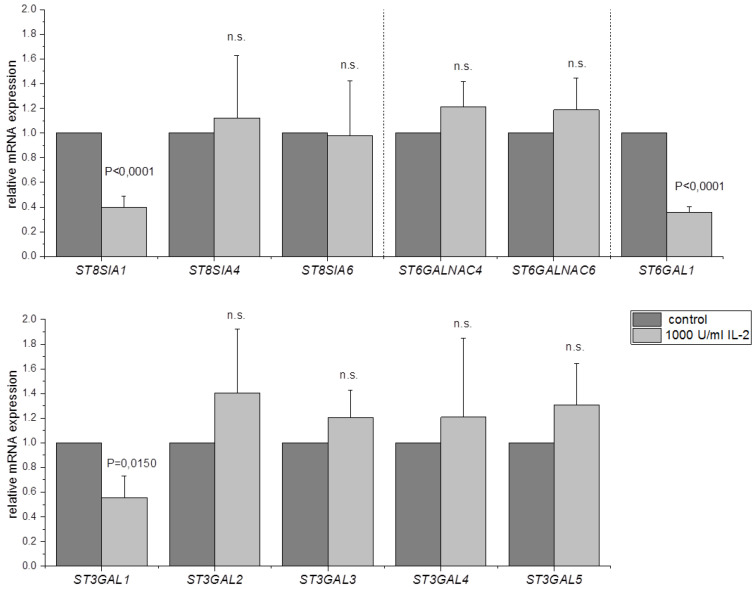
Expression of sialyltransferases in NK-92 cells after activation with IL-2. NK-92 cells were incubated without IL-2 for 24 h. Afterwards, cells were either left untreated (control) or treated with 1000 U/mL IL-2 for 4 h. cDNA was synthesized, and quantitative real-time PCR reactions were performed. Data were normalized to Beta-2 microglobulin (*B2M*) expression. Graphs show average mean ± SD of at least 3 independent experiments.

The activation of NK cells with IL-2 has an impact on the expression of many different genes [30]. To test whether IL-2 also changes the mRNA expression levels of the sialyltransferases, qPCR analyses were performed. NK-92 cells were incubated without IL-2 for 24 h and either left untreated or treated with 1000 U/mL IL-2 for 4 h (Figure 2) prior to qPCR and the expression levels of all sialyltransferases that are present in NK-92 cells were quantified. The data were normalized to Beta-2 microglobulin (*B2M*) as its expression is stable during IL-2 activation of NK cells [31]. Furthermore, the IL-2 mediated activation of NK cells was assessed by determination of the expression of IFN-γ via qPCR. IFN-γ is upregulated during IL-2 stimulation of NK cells [32], which was confirmed in this study (Supplementary Material Figure S3). In contrast, treatment of NK cells with IL-2 results in an approximately 60% downregulation of *ST8SIA1* and *ST6GAL1* compared to unstimulated cells. *ST3GAL1* was also downregulated and showed about 50% expression compared to untreated cells (Figure 2). All other sialyltransferases were not altered after activation with IL-2.

### 3.3. The Amount of α-2,6 Linked Sialic Acids and GD3 Is Changed after IL-2 Stimulation

**Figure 3:**
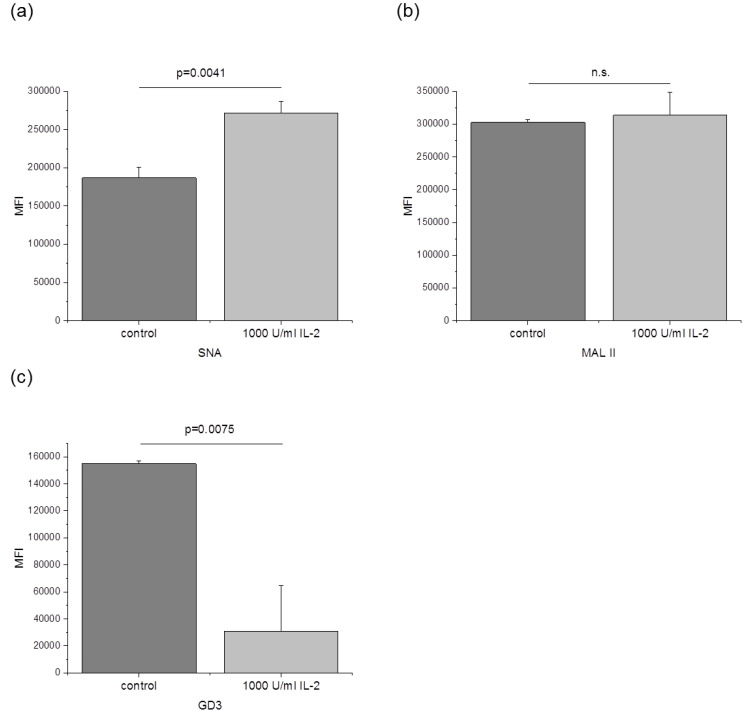
Analysis of NK cell sialylation by flow cytometry. NK-92 cells were incubated without IL-2 for 24 h and analyzed directly (control) or treated with 1000 U/mL IL-2 for additional 24 h. Cells were stained with (**a**) Sambucus Nigra Lectin (SNA), (**b**) Maackia Amurensis Lectin II (MAL II) or (**c**) anti-GD3 antibody and the mean fluorescence intensity (MFI) was measured by flow cytometry. Graphs show average mean ± SD of 3 (**a**,**b**) or 4 (**c**) independent experiments.

To investigate whether the activation with IL-2 also leads to changes of the sialylation, NK-92 cells were stained with Sambucus Nigra Lectin (SNA) and Maackia Amurensis Lectin II (MAL II) and analyzed by flow cytometry (Figure 3). SNA binds to sialic acids that are in an α-2,6 linkage with either Gal or GalNAc [30]. MAL II detects sialic acids attached via α-2,3 linkage to the Galβ1-3GalNAc structure [31]. The sialyltransferases *ST6GALNAC4* and *ST6GALNAC6* and *ST6GAL1* catalyzing the attachment of sialic acid via α-2,6 linkage could be detected in NK-92 cells (Table 2). qPCR analysis revealed that *ST6GAL1* was downregulated after IL-2 stimulation, while the expression of *ST6GALNAC4* and *ST6GALNAC6* was not changed (Figure 2). Interestingly, SNA signal was increased by about 45% after activation with IL-2, indicating a higher amount of α-2,6 linked sialic acids. Five sialyltransferases (*ST3GAL1-5*) that attach sialic acid via α-2,3 linkage are expressed in NK-92 cells (Table 2). *ST3GAL1* was downregulated after IL-2 treatment (Figure 2). Flow cytometry analysis showed that MAL II intensity remained unchanged after IL-2 stimulation. Therefore, no impact of the reduced *ST3GAL1* expression on the overall amount of α-2,3 sialic acids was confirmed. The qPCR data further indicate a reduced mRNA expression of *ST8SIA1* (Figure 2). This sialyltransferase is responsible for the synthesis of the disialoganglioside GD3 [32]. Accordingly, a staining with an anti-GD3 antibody was performed after stimulation with IL-2, and the cells were again analyzed by flow cytometry. GD3 signal was reduced by about 80% after treatment with IL-2 (Figure 3c). GD3 amount is decreased after activation with IL-2 most likely due to a reduced expression of *ST8SIA1* that was found to be downregulated in qPCR experiments.

### 3.4. PolySia and NCAM/CD56 Expression Is Increased after IL-2 Stimulation

**Figure 4:**
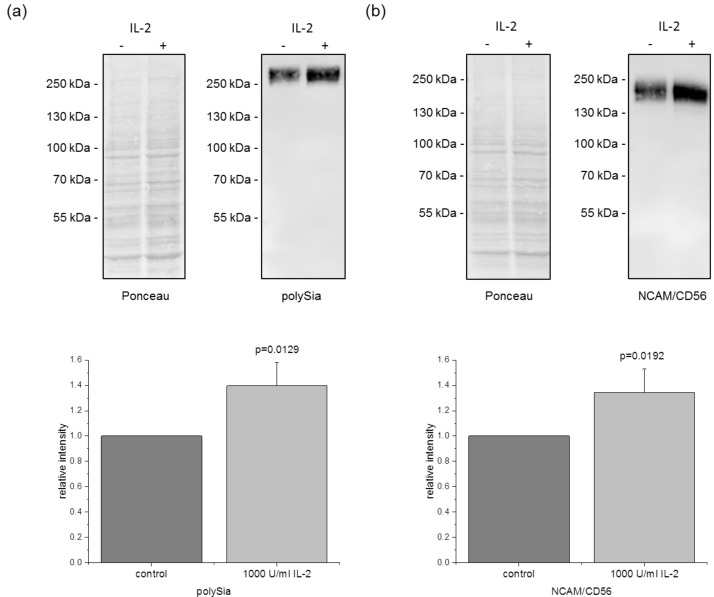
Western blot analysis of polySia and NCAM/CD56 after IL-2 activation. NK-92 cells were incubated without IL-2 for 24 h and analyzed directly (control) or treated with 1000 U/mL IL-2 for additional 24 h. Proteins were isolated, separated via SDS-PAGE and analyzed by Western blot. (**a**) Relative polySia intensity before and after treatment is shown. (**b**) For analysis of NCAM/CD56, samples were treated with endo-N-acetylneuraminidase E (endoNE) prior to separation. Graphs show average mean ± SD of 5 independent experiments and blots are representative.

As primary human NK cells, NK-92 cells express NCAM/CD56 on their surface, which has polySia attached. Therefore, Western blot analyses were performed to determine if the activation with IL-2 also has an impact on the amount of polySia. Upon treatment of NK cells with IL-2, the polySia signal was increased by about 40% (Figure 4a), whereas the mRNA expression of *ST8SIA4*, which catalyzes the polySia synthesis, was unchanged (Figure 2). Hence, the expression of NCAM/CD56 was also analyzed by Western blot. PolySia has a high molecular weight with different chain lengths, which can result in a blurred NCAM/CD56 signal in the Western blot. To overcome this issue, samples were treated with the sialidase endoNE prior to NCAM/CD56 analysis, which removes polySia. The NCAM/CD56 signal was increased approximately to the same degree as the polySia signal (Figure 4b) indicating that the higher amount of polySia is a result of a higher expression of the carrier molecule NCAM/CD56 after activation with IL-2.

## 4. Discussion

PCR analyses revealed that *ST8SIA1*, *ST8SIA4*, *ST8SIA6*, *ST6GALNAC4*, *ST6GALNAC6*, *ST6GAL1* and *ST3GA1-5* were expressed in the human NK cell lines NK-92, NKL and KHYG-1 and primary NK cells. The two sialyltransferases ST8Sia II and ST8Sia IV synthesize polySia, and only the expression of the ST8Sia IV related gene *ST8SIA4* was found in human NK cells [22,33]. Similar observations were made in the present study, where the expression of *ST8SIA4* was found in all NK cell lines and primary NK cells, but *ST8SIA2* was not expressed. Interestingly, *ST8SIA4* was also detected in the NKL cell line in this study even though these cells lack the expression of the acceptor molecule NCAM/CD56 [29] and had no polySia. However, *ST8SIA4* expression could be also found in other cell types that do not express NCAM/CD56 [33]. It remains unclear whether this sialyltransferase is functionless in NKL cells without NCAM/CD56. The primary NK cells expressed the same sialyltransferases as the NK cell lines. In addition, in NK cells from 2 of the 3 donors, an expression of *ST6GAL2* and *ST3GAL6* was found. One of the donors expressing *ST6GAL2* and *ST3GAL6* was male and the other was female. Due to these data and the low number of donors, we cannot conclude that age or gender has an influence on the expression of *ST6GAL2* and *ST3GAL6*. Nevertheless, further cohort studies are required to determine how the expression of these sialyltransferases is regulated in primary NK cells and why they are only present in certain donors. Because primary NK cells are very heterogeneous, the well-characterized NK cell line NK-92 was used to study the effect of IL-2 incubation on the sialyltransferase expression. Findings from these experiments can then serve as a basis for further clinical studies. After IL-2 stimulation, the mRNA expression levels of *ST8SIA1*, *ST6GAL1* and *ST3GAL1* were reduced. Similar observations were made in murine T cells, where different glycosyltransferases including *ST6GAL1* and *ST3GAL1* were downregulated after stimulation with IL-2 [34].

Flow cytometric analyses revealed that the amount of α-2,6 linked sialic acids is increased in NK-92 cells stimulated with IL-2. Together with the results of the qPCR analysis this seems not to be a result of a higher sialyltransferases expression. Indeed, *ST6GAL1* was downregulated. It is known, that IL-2 changes the expression of many different receptors [18,19,20]. Therefore, alterations in the expression of cell surface proteins that are differentially sialylated might be the cause of the higher amount of α-2,6 linked sialic acids. The expression of *ST3GAL1* was also reduced after IL-2 stimulation. However, the MAL II staining showed no difference between treated and untreated NK cells. Other sialyltransferases might fulfill their task for most substrates leaving the overall α-2,3 sialylation unchanged. Apart from sialyltransferases, alterations in the sialic acid biosynthesis and in the transport to the Golgi would also have an impact on cell sialylation. Moreover, human cells express 4 types of neuraminidases (NEU1-4) that remove sialic acids from glycan structures [35]. Thus, a different neuraminidase activity after activation might also contribute to the observed changes in sialylation of NK cells.

Nevertheless, it cannot be excluded that the reduced expression of *ST6GAL1* and *ST3GAL1* is important for regulating the sialylation of certain molecules. Glycosylation can influence the half-life and abundance of proteins. For example, the sialylation by ST6Gal I is important for the stability of the adhesion molecule ICAM-1 [36]. Sialylation can also have an impact on the receptor function. The activating receptor CD244 on NK cells has a stronger ligand binding after desialylation and IL-2 stimulation changes its glycosylation, which contributes to a higher NK cell activity [37]. However, the sialyltransferases involved in this process are still unknown. The downregulation of *ST6GAL1* and *ST3GAL1* after IL-2 treatment, which were observed in our study, might be one reason for the changes in the glycosylation of CD244. Therefore, identifying acceptor molecules for ST6Gal I and ST3Gal I in NK cells is important for understanding the role of sialylation on NK cell activation.

*ST8SIA1* was also downregulated after activation with IL-2. This sialyltransferase synthesizes the disialoganglioside GD3 [38], and the amount of GD3 was found to be reduced after IL-2 treatment. GD3 is mainly studied in tumor cells, because it is expressed at high levels in different tumor entities [39]. Nevertheless, GD3 is expressed on different immune cell types but at lower levels [40]. It is known, that Siglec-7 binds to GD3 on tumor cells and is masked by a yet unknown ligand [33,41]. Therefore, it might be possible that GD3 on NK cells plays a role in masking Siglec-7 and that this masking is regulated via *ST8SIA1* expression. Moreover, as the GD3 level is markedly decreased after IL-2 stimulation, GD3 might be used as a marker for non-activated NK cells.

NK cells have polySia on their cell surface, which is a nearly unique modification of NCAM [22]. Western blot analysis revealed that polySia was increased after IL-2 activation, and further analysis showed that NCAM/CD56 signal was also increased. This is in accordance with studies from Drake et al. [42] who analyzed IL-2 activated primary human NK cells by flow cytometry and also reported an increased NCAM/CD56 and polySia signal. The higher amount of polySia is probably a direct effect of the higher amount of NCAM/CD56 as the present study shows that *ST8SIA4* is not influenced by IL-2. The function of NCAM/CD56 on NK cells is still under investigation, but some studies suggest a role in NK cell development, motility and pathogen recognition [43,44]. Based on these data, it is postulated that an upregulation of NCAM/CD56 might therefore be important for the correct function of NK cells.

To conclude, we were able to characterize primary NK cells as well as NK cells lines based on their sialyltransferase expression pattern. Furthermore, the expression of *ST8SIA1*, *ST6GAL1* and *ST3GAL1* seems to be influenced by IL-2 because these sialyltransferases were downregulated after IL-2 stimulation. After incubation with IL-2 the sialylation of NK-92 cells was changed, more precisely α-2,6 linked sialic acids were increased and GD3 was decreased. Besides, a higher amount of polySia could be found after IL-2 activation, most likely due to an increase in the expression of the carrier molecule NCAM/CD56. Therefore, IL-2 was shown to have an impact on the sialyltransferase expression and the sialylation of NK cells.

## Figures and Tables

**Table 2:** Expression of sialyltransferases in human NK cells.

	NK-92 (*n* = 3)	NKL (*n* = 3)	KHYG-1 (*n* = 3)	Primary NK Cells (From 3 Donors)
ST8SIA1	+	+	+	3/3
ST8SIA2	-	-	-	-
ST8SIA3	-	-	-	-
ST8SIA4	+	+	+	3/3
ST8SIA5	-	-	-	-
ST8SIA6	+	+	+	3/3
ST6GALNAC1	-	-	-	-
ST6GALNAC2	-	-	-	-
ST6GALNAC3	-	-	-	-
ST6GALNAC4	+	+	+	3/3
ST6GALNAC5	-	-	-	-
ST6GALNAC6	+	+	+	3/3
ST6GAL1	+	+	+	3/3
ST6GAL2	-	-	-	2/3
ST3GAL1	+	+	+	3/3
ST3GAL2	+	+	+	3/3
ST3GAL3	+	+	+	3/3
ST3GAL4	+	+	+	3/3
ST3GAL5	+	+	+	3/3
ST3GAL6	-	-	-	2/3

Data are derived from PCR analysis (see Supplementary Material Figure S2).

## Supplementary Materials

The following are available online at https://www.mdpi.com/2077-0383/9/6/1816/s1. Table S1: Human sialyltransferases. Figure S1: Gating strategy for flow cytometry. Figure S2: Sialyltransferase expression in NK cells. Figure S3: Expression of IFN-γ in NK-92 cells after activation with IL-2.
