# Supplementary material for: Sialylation of Human Natural Killer (NK) Cells Is Regulated by IL-2
Source: J Clin Med. 2020 Jun 11;9(6):1816. doi: 10.3390/jcm9061816 (PMC7356531; doi:10.3390/jcm9061816)
Supplement: Supplementary file 1 [file jcm-09-01816-s001.pdf]

Table S1. Human sialyltransferases (edited from Takashima et al., 2002 [8]).

Enzyme	Amino acid length	Gene	Chromosomal localization	Substrate
ST8Sia I	356	*ST8SIA1*	12p12.1-p11.2	GM3
ST8Sia II	375	*ST8SIA2*	15q26	*N*-glycan on NCAM
ST8Sia III	380	*ST8SIA3*	18q21	NeuAcα2,3Galβ1,4GlcNAc-
ST8Sia IV	359	*ST8SIA4*	5q21	*N*-glycan on NCAM
ST8Sia V	376	*ST8SIA5*	18q12.1-q12.3	GM1b, GT1b, GD1a, GD3
ST8Sia VI	398	*ST8SIA6*	10p13	NeuAcα2,3(6)Gal-
ST6GalNAc I	600	*ST6GALNAC1*	17q25	GalNAcα1,*O*-Ser/Thr
ST6GalNAc II	374	*ST6GALNAC2*	17q25	Galβ1,3GalNAcα1,*O*-Ser/Thr
ST6GalNAc III	305	*ST6GALNAC3*	1p31.1-p31.2	NeuAcα2,3Galβ1,3GalNAc-(Lipid)*
ST6GalNAc IV	302	*ST6GALNAC4*	9q34	NeuAcα2,3Galβ1,3GalNAc-(Protein)*
ST6GalNAc V	336	*ST6GALNAC5*	1p31.1	GM1b
ST6GalNAc VI	299	*ST6GALNAC6*	9q34	GM1b, GT1b, GD1a
ST6Gal I	406	*ST6GAL1*	3q27-q28	Galβ1,4GlcNAc-
ST6Gal II	529	*ST6GAL2*	2q11.2-q12.1	Galβ1,4GlcNAc-
ST3Gal I	340	*ST3GAL1*	8q24.2	Galβ1,3GalNAc-(Protein)*
ST3Gal II	350	*ST3GAL2*	16q21–22.3	Galβ1,3GalNAc-(Lipid)*
ST3Gal III	375	*ST3GAL3*	1pter-p32.3	Galβ1,3(4)GlcNAc-
ST3Gal IV	329	*ST3GAL4*	11q23-q24	Galβ1,4(3)GlcNAc-
ST3Gal V	362	*ST3GAL5*	2p24.3-p24.1	Lac-Cer
ST3Gal VI	331	*ST3GAL6*	3p21.1-q13.2	Galβ1,4GlcNAc-

* preferential but not specific substrate. Lac-Cer (lactosylceramide), GM (monosialoganglioside), GD (disialoganglioside), GT (trisialogangliosid).

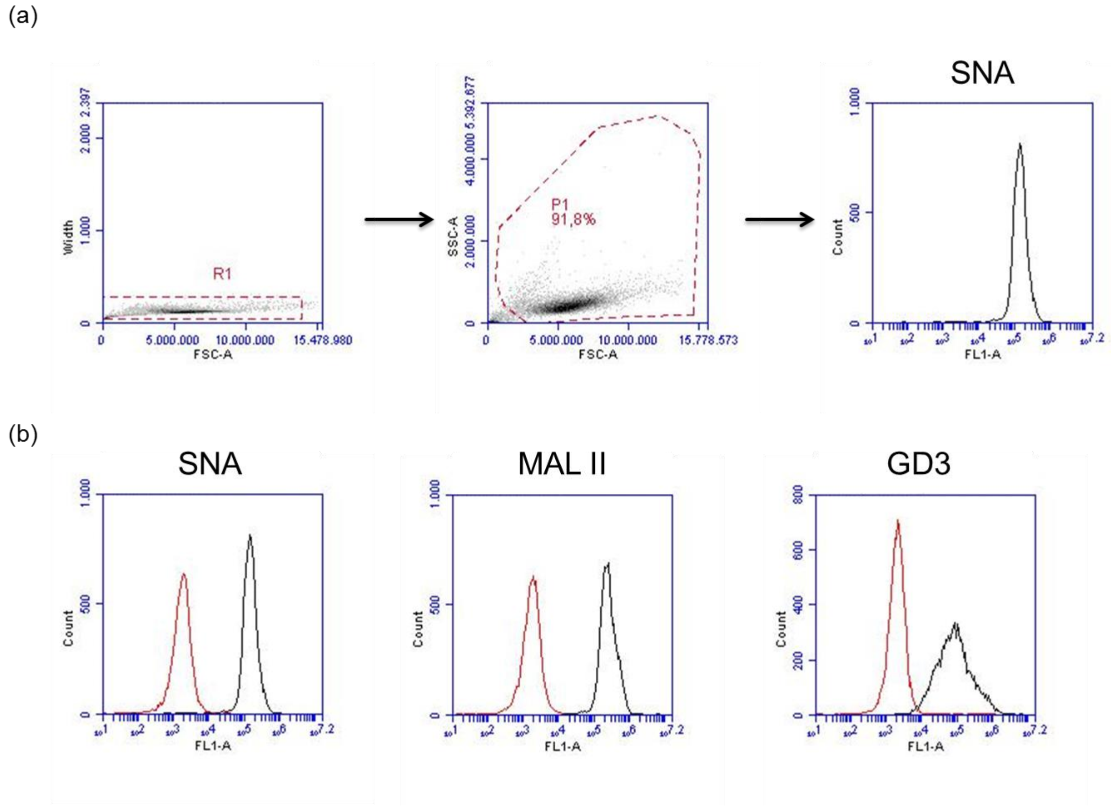

Figure S1. Gating strategy for flow cytometry. (**a**) Forward scatter (FSC) area against width was used for doublet discrimination whereas FSC versus sideward scatter (SSC) was used for removal of debris. 10,000 events were recorded in the P1 gate and analyzed in the FL1 channel. A representative histogram for Sambucus Nigra Lectin (SNA) is shown as an example. (**b**) Representative histograms from the staining with SNA, Maackia Amurensis Lectin II (MAL II) or GD3 are shown in black. The red histogram represents the streptavidin (for SNA and MAL II) or secondary antibody control (for GD3).

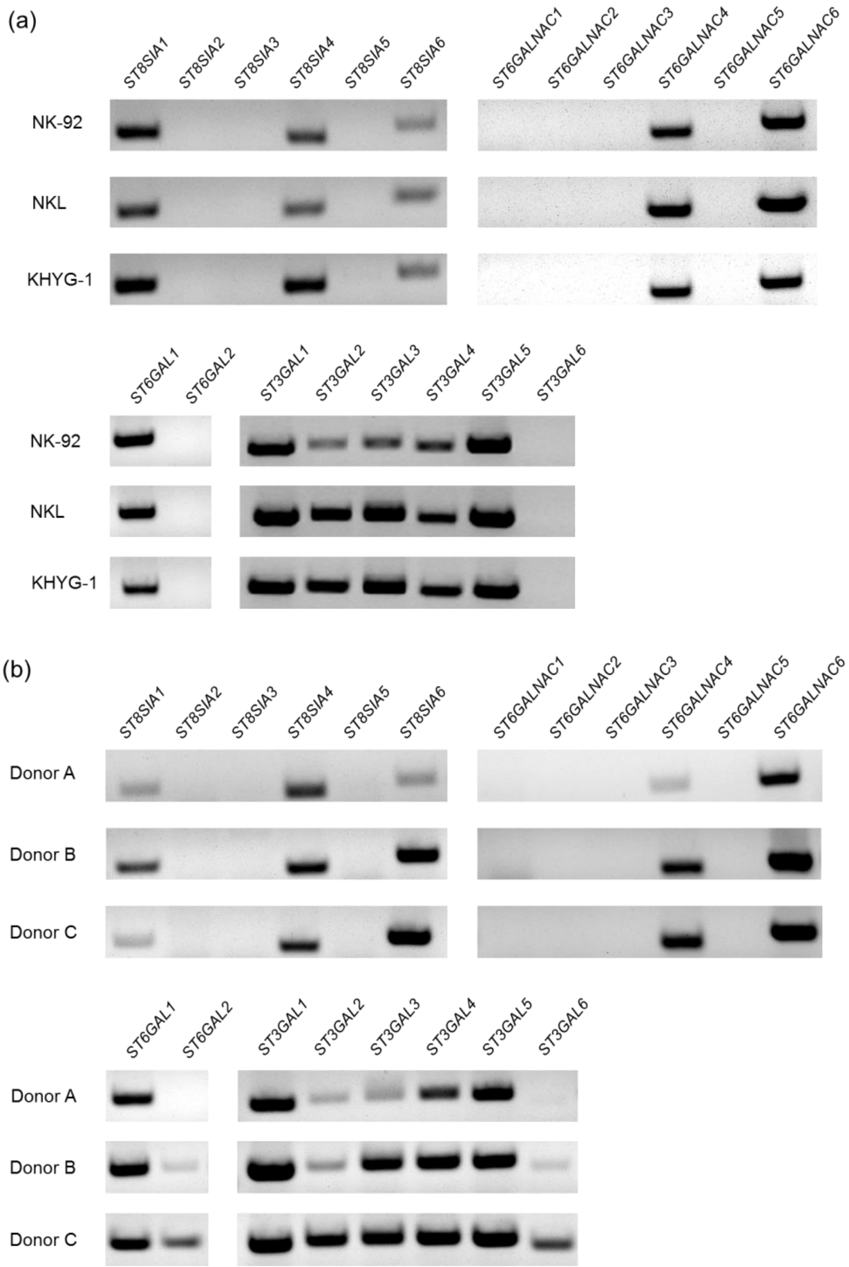

Figure S2. Sialyltransferase expression in NK cells. RNA was isolated from the human NK cell lines NK-92, NKL, KHYG-1 and primary NK cell. cDNA was synthesized and PCR reactions were performed with primer pairs for all 20 human sialyltransferases. (**a**) Representative agarose gels are shown from 3 independent experiments performed with cDNA derived from the NK cell lines. (**b**) Gels from 3 independent experiments performed with cDNA derived from 3 healthy blood donors are shown. For product sizes see Table 1.

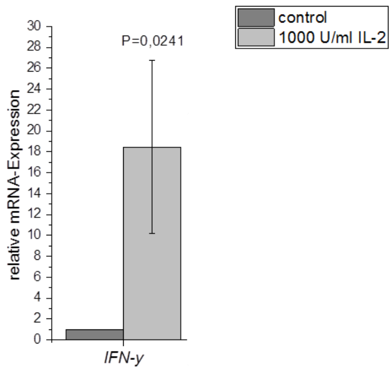

Figure S3. Expression of *IFN-γ* in NK-92 cells after activation with IL-2. NK-92 cells were incubated without IL-2 for 24 h. Afterwards, cells were either left untreated (control) or treated with 1000 U/ml IL-2 for 4 h. cDNA was synthesized and quantitative real-time PCR reactions were performed. Data were normalized to Beta-2 microglobulin (*B2M*) expression. Graphs show average mean ± SD of 4 independent experiments.
